# Fluorescent Self‐Healing Elastomers with Triple Dynamic Bonds for 2D/3D Printed Information Encryption

**DOI:** 10.1002/smsc.202500091

**Published:** 2025-07-30

**Authors:** Dai Yang, Qingyong Tian, Jingyang Li, Xiaoqing Sui, Shuiren Liu, Xiaoguang Hu, Qingqing Sun, Linlin Zhang, Mingjun Niu, Xuying Liu, Weijing Yao

**Affiliations:** ^1^ School of Materials Science and Engineering Henan Institute of Advanced Technology Zhengzhou University Zhengzhou 450001 P. R. China

**Keywords:** 2D/3D printing, information encryption and anticounterfeiting, metal coordination, self‐healing elastomers, up/down‐conversion nanoparticles

## Abstract

The development of novel optical self‐healing materials holds significant importance for applications in anticounterfeiting and information encryption, but remains a formidable challenge. This study presents a fluorescent self‐healing material designed for 2D/3D printing anticounterfeiting applications, exhibiting outstanding properties such as high transmittance, excellent mechanical strength, and remarkable self‐healing efficiency. The triple dynamic bond networks provide robust mechanical and self‐healing capabilities, with the polymer demonstrating a tensile strength of 26.9 MPa, an elongation at break of 1400%, toughness of 149.4 MJ m^−3^, and a self‐healing efficiency of 97%. When incorporated with core–shell nanoparticles, the polymer forms a fluorescent elastomer capable of triple‐mode up/down conversion fluorescence emission. This material can be easily customized via 2D/3D printing to create the desired shapes, and its self‐healing property allows for the combination of various configurations, thereby facilitating the encryption of multiple information applications. This study presents an effective protocol for synthesizing fluorescent self‐healing materials, further advancing their potentials for use in information encryption and fluorescence‐based anti‐counterfeiting.

## Graphical abstract

A self‐healing elastomer with triple dynamic bonds and 2D/3D printing capability is prepared in this work, and synthesized triple‐mode core–shell fluorescent nanoparticles are introduced to enable bright fluorescence under 254, 808, and 980 nm laser irradiation. 2D/3D printing is found to efficiently and conveniently customize patterns or shapes to fabricate information encryption and anticounterfeiting devices.

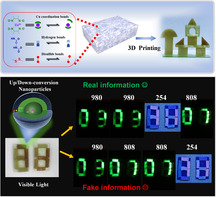

## 1 Introduction

Fluorescence anticounterfeiting patterns are widely used in banknotes, packaging labels, and genuine medicine anticounterfeiting due to their cost‐effectiveness and high information encryption capacity.^[^ ^1^ ^]^ Traditional fluorescent materials primarily encompass organic fluorescent dyes, inorganic perovskite nanocrystals, and carbon dots (CDs).^[^ ^2^ ^]^ The fluorescence emitted by these materials produces a wide range of colors when exposed to laser irradiation.^[^ ^3^ ^]^ In addition, core–shell luminescent nanomaterials doped with rare‐earth ions have distinctive optical properties, resulting in multicolor emission peaks, narrow emission spectrum, and good photostability.^[^ ^4^ ^]^ However, the effectiveness of fluorescent anticounterfeiting patterns is often compromised by mechanical damage^[^ ^5^ ^]^ during practical use, limiting their application. Biomimetic self‐healing materials that can autonomously repair damage without external intervention have attracted significant attention in recent years, and these materials are expected to enhance longevity,^[^ ^6^ ^]^ safety, and environmental impact across various applications.^[^ ^7^ ^]^ Notably, the combination of core–shell luminescent nanomaterials with the self‐healing material can effectively improve the durability of optical anti‐counterfeiting patterns, improve information concealment security, and broaden their application range.^[^ ^8^ ^]^ In addition, developing self‐healing luminescent materials with high transparency is critical for fabricating advanced data encryption devices. These materials are invisible under visible light and contain highly encrypted information, and are capable of responding to external stimuli for color switching.

Current self‐healing strategies for polymer materials can be generally divided into two categories (extrinsic‐ and intrinsic) based on their structural characteristics and chemical composition.^[^ ^9^ ^]^ The extrinsic category, lacking an external healing agent, exhibits a constrained repair efficacy, restricting its extensive application.^[^ ^10^ ^]^ Comparatively, intrinsic self‐healing capability can be repeatedly achieved through the recombination of dynamic covalent bonds, such as Diels Alder, disulfide bonds, or noncovalent interactions, including hydrogen bonds,^[^ ^11^ ^]^ ionic interactions, and metal ligands coordination, along with chain exchange and entanglement.^[^ ^12^ ^]^ Current research has focused on the exploration of intrinsic self‐healing materials that rely on dynamic covalent bonds or noncovalent interactions.^[^ ^13^ ^]^ However, simultaneously achieving superior mechanical properties and dynamic healing in self‐healing material poses a significant challenge.^[^ ^14^ ^]^ Weaker interactions can expedite chain exchange and enhance healing efficiency, often resulting in materials with softer and more viscoelastic characteristics. By contrast, stronger interactions tend to attenuate dynamic bonding and chain recombination exchange, leading to a reduction in healing efficiency while improving the mechanical properties of the materials. One strategy involving the fine‐tuning of metal ions and ligands in coordination bonds, characterized by unique noncovalent interactions between metal ions and their surrounding organic ligands, can balance the mechanical and self‐healing properties of self‐healing polymers. For example, Lai et al. designed and synthesized a self‐healing polymer featuring a unique coordination system by combining polydimethylsiloxane with the alternating ligands of Zn complexes, which exhibited high association constants.^[^ ^15^ ^]^ By precisely controlling the stoichiometric ratio of metal ions and ligands, the material was endowed with excellent healing efficiency and strong mechanical properties (toughness: 29.3 MJ m^−3^, self‐healing efficiency: 98.9 ± 1.9%). Xu et al. synthesized a supramolecular polyurethane (PU) elastomer by introducing 2‐ureido‐4[1H] pyrimidinone radicals to coordinate with Zn^2+^, inducing a superior tensile strength of 14.15 MPa, along with outstanding toughness (47.57 MJ m^−3^) and Young's modulus (146.92 MPa).^[^ ^16^ ^]^ In addition, the complementarity of covalent and noncovalent bonds can be exploited to balance the mutual constraints between self‐healing and mechanical properties.^[^ ^17^ ^]^ The introduction of disulfide bonds can endow the material with more dynamic bonds, resulting in stronger repair capabilities.^[^ ^18^ ^]^ In general, introducing highly adjustable metal coordination bonds and multiple dynamic bonds into the synthesis of materials serves as a promising strategy to achieve exceptional mechanical strength and efficient self‐healing capabilities.

Fluorescent self‐healing materials offer broad application prospects in fields such as information anticounterfeiting, information encoding, and encryption.^[^ ^19^ ^]^ However, the process of patterning self‐healing elastomers remains relatively intricate, and the preparation of encoder components is similarly complex, posing significant challenges to the rapid and effective customization of anticounterfeiting devices. 2D/3D printing technologies provide a convenient approach for patterning self‐healing materials. 3D printing, an additive manufacturing technique, facilitates the rapid fabrication of intricate 3D architectures, according to specific requirements.^[^ ^20^ ^]^ The utilization of 3D printing offers significant advantages in terms of unrestricted customization, streamlined production process, and enhanced cost control.^[^ ^21^ ^]^ This process encompasses advanced additive manufacturing methods, including selective laser sintering, extrusion molding techniques such as fused deposition modeling (FDM) and direct ink writing, as well as stereolithography.^[^ ^22^ ^]^ Among these, FDM technology has been adopted across diverse fields due to its simplistic structure, cost‐effective maintenance process, and exceptional material utilization rate.^[^ ^23^ ^]^ However, FDM still faces several challenges. First, its interlayer bonding force is relatively weak, resulting in inadequate mechanical properties of the printed samples. Second, the printing of large objects frequently requires the design and implementation of support structures, which can be cumbersome and may damage the printed objects during their removal.^[^ ^24^ ^]^ These factors significantly limit the widespread application of 3D printing in various fields. Self‐healing materials can enhance the interlayer adhesion of printed samples through repair. Assembling large objects using building blocks can eliminate the need for designing support structures and avoid the subsequent need for their removal. In addition, 3D printing can rapidly and effectively customize information encoding devices, addressing the issue of elastomer patterning.

In this work, we proposed a 2D/3D printed self‐healing PU film with triple‐mode fluorescence response. The incorporation of polytetramethylene ether glycol (PTMEG) as the soft segment, along with the addition of isophorone diisocyanate (IPDI, mixture of isomers) and 2‐hydroxyethyl disulfide (HDES), resulted in the formation of hydrogen and dynamic disulfide bonds in the film. The combination of dimethylglyoxime (DMG) and anhydrous copper (II) chloride (CuCl_2_) also produced highly adjustable metal coordination bonds, improving the mechanical and self‐healing properties of the material. The obtained comprehensive elastomer exhibited good mechanical strength and high transparency, with a tensile strength of 26.9 MPa, toughness of 149.4 MJ m^−3^, and transmission rate of >97% at 560 nm. The synthesized triple‐mode core–shell fluorescent nanoparticles were uniformly dispersed in the polymer matrix, enabling unique strong fluorescence upon excitation at 254, 808, and 980 nm. Through 3D printing technology, fluorescence devices with encrypted information could be customized with significant freedom and rapid preparation. The fluorescent elastomer was also dissolved in *N*,*N*‐dimethylformamide (DMF) to produce a fluorescent ink suitable for screen‐printing 2D anticounterfeiting patterns.

## 2 Results and Discussion

**Figure 1:**
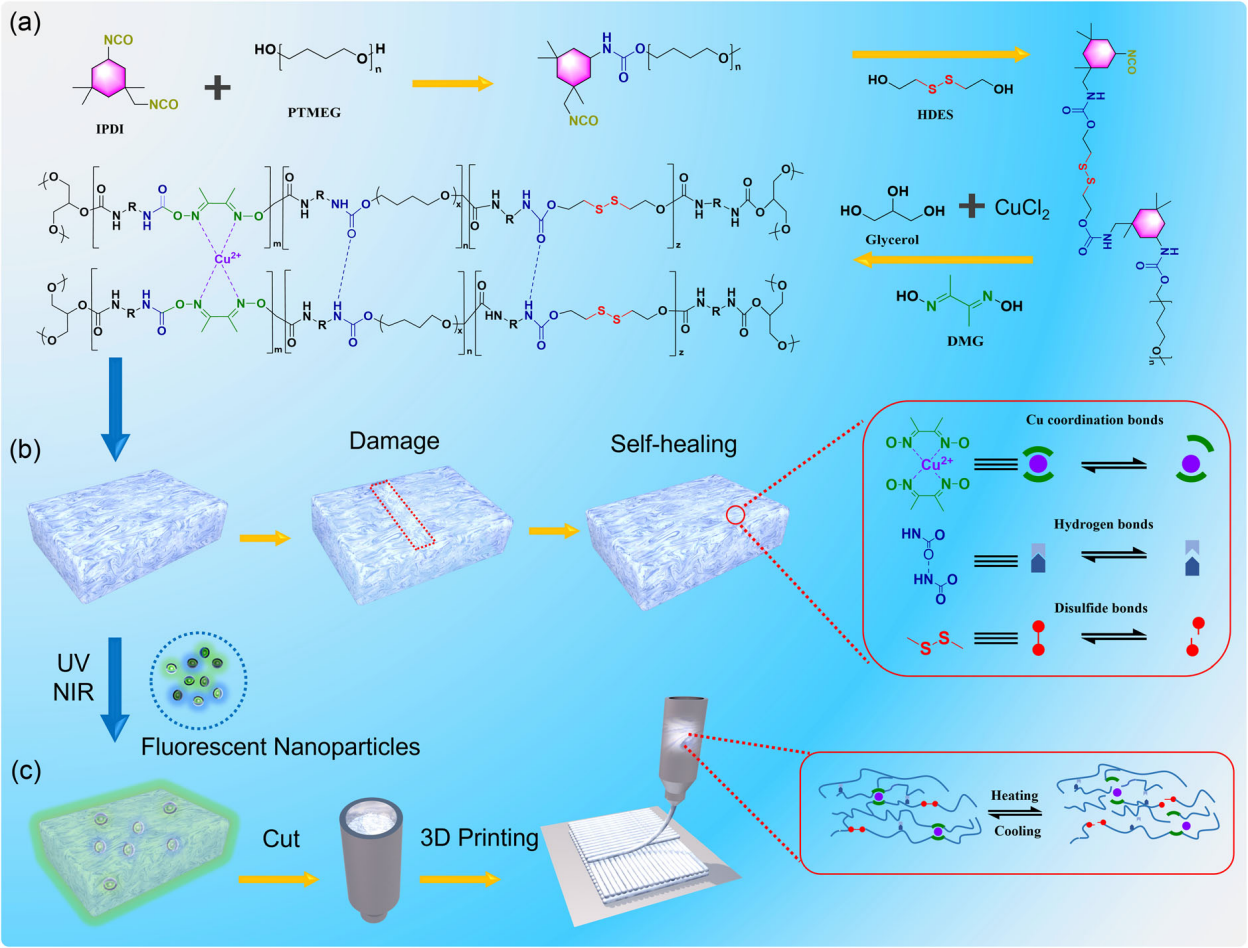
Molecular design of PUCS polymers and synthesis methods of fluorescent elastomers. a) The synthetic route and molecular structure of PUCS elastomer. b) Schematic structure of PUCS for self‐healing process, including coordination bonds, hydrogen bonds, and disulfide bonds. c) Preparation of fluorescent elastomers and 3D printing process of PUCS.

The synthesis of the self‐healing polyurethane elastomer (PUCS) was achieved through a prepolymerization method, as shown in **Figure** **1**a. Flexible PTMEG (*M*_n_ ≈ 1000) with pliable chains served as the soft segment, which facilitated chain mobility. Subsequently, the prepolymer was formed by reacting IPDI as the hard segment, due to its excellent light resistance and nonyellowing properties. The significant asymmetric lipid ring structure of IPDI effectively inhibited crystallization and provided chain mobility, facilitating the preparation of PU with high‐transparency and favorable self‐healing properties. The presence of HDES and DMG as chain extenders was instrumental in this process. HDES facilitated the formation of numerous dynamic disulfide bonds, while the methyl group in DMG hindered crystallization and enhanced chain mobility. The nitrogen atom of the adjacent oximide group readily formed coordination bonds with Cu^2+^, leading to the formation of metal coordination complexes. The PUCS material incorporated three types of dynamic bonds, including hydrogen, disulfide, and Cu^2+^ coordination bonds, which collectively contributed to its exceptional healing properties and mechanical performance (Figure 1b). Subsequently, a multimodal fluorescent elastomer was synthesized through the incorporation of three‐modal core–shell fluorescent nanoparticles during the preparation process of PUCS. Additionally, the presence of multiple dynamic bonds in the elastomer enabled high temperature FDM 3D printing, as illustrated in Figure 1c

**Figure 2:**
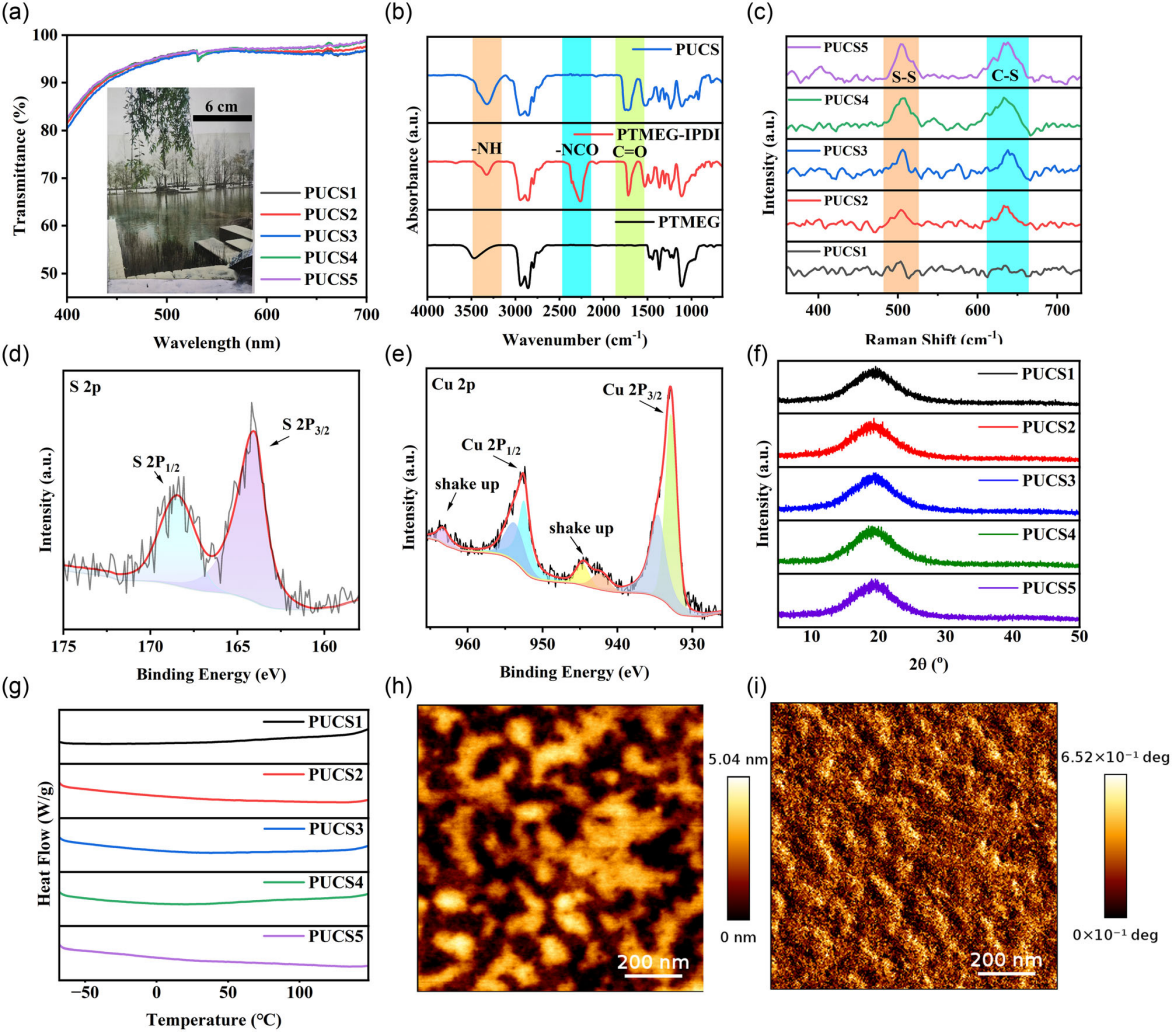
Structural characterization of PUCS. a) Transmission spectra and images of PUCS polymers. b) FTIR spectra of PTMEG, PTMEG–IPDI, and PUCS prepolymer. c) Raman spectra of PUCS. d,e) XPS spectra of PUCCS S 2*p* (d) and Cu 2*p*. f) XRD patterns of PUCS elastomers. g) DSC curves recorded of PUCS. h) AFM height image and i) phase image of PUCS4 polymers.

The transmittance test confirmed the optical transparency of the sample. A baseline calibration of the light transmission test using glass demonstrated that the synthesized PUCS elastomer exhibited a high light transmission rate (>97%) at 560 nm, nearly equivalent to that of transparent glass. Furthermore, this elevated level of light transmission was consistently observed across all tested samples. As shown in **Figure** **2**a, the sample allowed for the precise examination of even intricate details in the background image. Fourier transform infrared (FTIR) analysis revealed the disappearance of the peaks at 3465 cm^−1^ for PTMEG and 2262 cm^−1^ for the PTMEG–IPDI prepolymer. Furthermore, the presence of the —NH peak at 3326 cm^−1^ and C=O peak at 1725 cm^−1^ in the synthetic product validated the complete reaction of N=C=O (Figure 2b and S1, Supporting Information). The N—O bond at 987 cm^−1^ experienced a shift to 972 cm^−1^ following the addition of copper ions (Figure S2, Supporting Information), providing initial evidence for the presence of a copper complex.^[10a]^ The peaks observed at 506 and 632 cm^−1^ in the Raman spectrum were indexed to the vibrational modes of ν(S—S) and ν(C—S), respectively, as shown in Figure 2c. Notably, the intensity of these peaks showed a proportional increase according to disulfide bond content, indicating the successful integration of disulfide bonds into the elastomer. The presence of S, C, N, and O elements and copper complexes in PUCCS was assessed by X‐ray photoelectron spectroscopy (XPS) (Figure 2d,e, and S3, Supporting Information). The S 2*p* signal exhibited two distinct two peaks at S 2*p*_1/2_ (168.47 eV) and S 2*p*_3/2_ (164.00 eV), respectively. The peak observed at 932.85 eV in the Cu 2*p* spectrum (Figure 2e) corresponded to a copper complex, while the oscillating peaks ranging from 940.5 to 946.5 and 963.25 eV indicated the presence of copper ions at specific distribution sites. The C 1*s* spectrum (Figure S3b, Supporting Information) contained four distinct peaks representing the bonds of C—C (284.80 eV), C—O—C (286.16 eV), C—O (288.11 eV), and C=O (289.36 eV), respectively. In the N 1*s* spectrum (Figure S3c, Supporting Information), two prominent peaks were observed at 399.81 and 400.74 eV, corresponding to C—N and N—H bonds, respectively. In addition, significant peaks related to C—O (532.13 eV) and C=O (533.77 eV) bonds were observed in the O 1*s* spectrum (Figure S3d, Supporting Information). The presence of amorphous structures was crucial for achieving high self‐healing efficiency, as the crystallization process could hinder polymer chain mobility.^[^ ^25^ ^]^ Therefore, X‐ray diffraction (XRD) and differential scanning calorimetry (DSC) techniques were employed to characterize the noncrystalline structure of the samples (Figure 2f,g). XRD analysis revealed a prominent and broad peak at 19°, indicative of an amorphous polymer structure. Similarly, the DSC curve revealed the absence of any crystallization or melting peaks, providing further evidence for the amorphous structure of the PUCS sample. The molecular weight and polydispersity index (PDI) of the samples were analyzed by gel permeation chromatography (GPC), as shown in Table S2, Supporting Information. The height and phase images obtained from atomic force microscopy (AFM) analysis (Figure 2h,i and S4, Supporting Information) revealed a distinct nanoscale microphase separation structure in the sample, which could be ascribed to the inherent resistance of the IPDI asymmetric alicyclic structure to change, as well as the relatively limited flexibility of PTMEG chain segments.^[10a]^ This led to segregation between the hard and soft phases. The cross‐linking of DMG, HDES, and IPDI enhanced the level of microphase separation, and the incorporation of soft segments contributed to its good tensile characteristics.^[^ ^26^ ^]^ The aforementioned results demonstrated the substantial presence of copper complexes, disulfide bonds, and hydrogen bonds in PUCS. Therefore, we successfully synthesized highly transparent self‐healing elastomers featuring triple dynamic bonds.

**Figure 3:**
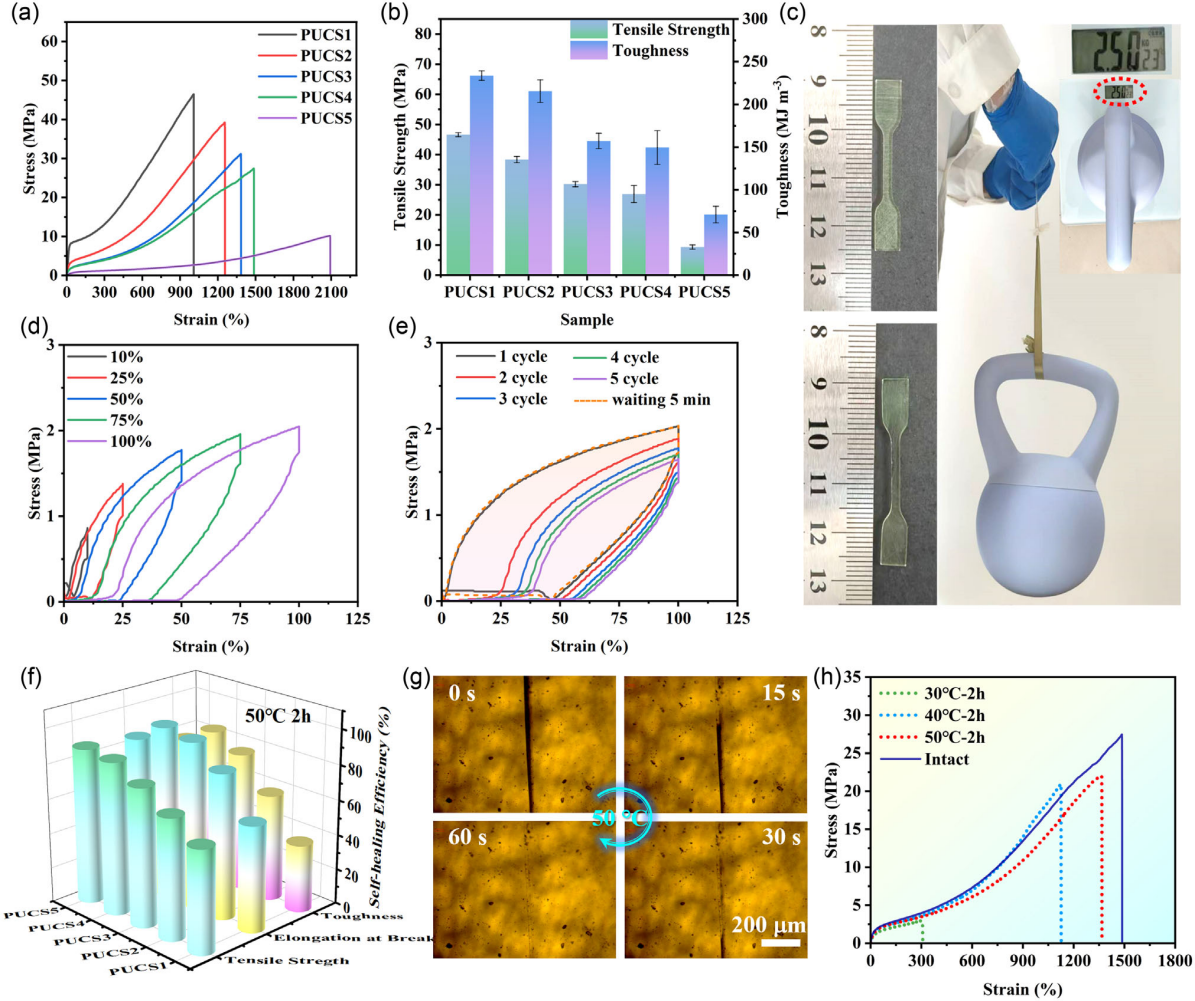
Mechanical performance and self‐healing properties of PUCS elastomers. a) Stress–strain curves of PUCS elastomers. b) The comparative histogram of toughness and tensile strength among different samples of PUCS. c) The photograph dumbbell‐shaped sample (0.14 g) lifts up 2.5 kg weight (right), the sample before (top) and after (bottom) lifting in the left. d) Sequential cyclic tensile curves of PUCS at different strains without waiting time between two consecutive loadings. e) The consecutive cyclic loading–unloading curves of PUCS with a strain of 100%. f) Self‐healing efficiency of PUCS after recovery at 50 °C for 2 h. g) The scratch self‐healing optical microscopy image of PUCS4 at 50 °C. h) Stress–strain curves of intact and damaged PUCS4 self‐healing at 30, 40, and 50 °C for 2 h.

Stress–strain tests were subsequently conducted to evaluate the mechanical properties of the samples. As shown in **Figure** **3**a,b and Table S2, Supporting Information, PUCS1 in the absence of HDES exhibited the highest tensile strength (46.6 MPa) and toughness (233.6 MJ m^−3^). The reduction in molar ratio of Cu^2+^/HDES led to a simultaneous decrease in both tensile strength and toughness, but with an enhancement in elongation at break. PUCS5 exhibited a tensile strength of 9.3 MPa, elongation at break of 2020%, and toughness of 70.9 MJ m^−3^. The tensile strength and toughness of PUCS5 experienced a significant decrease compared to PUCS1, and comparatively, the elongation at break was twice that of PUCS1. This could be ascribed to the formation of coordination bonds, which enhanced the rigidity of the molecular network and improved the cross‐linking strength between the chain segments, resulting in elevated elastomer strength and toughness.^[^ ^27^ ^]^ However, this also hindered chain segment movement and reduced sample elongation at break. Disulfide bonds acted as weak crosslinked bonds that facilitated chain segment movement. Therefore, as the content of coordination bonds decreased and disulfide bonds increased, an obvious increase in elongation at break was observed, along with a reduction in sample strength and toughness. Comprehensively, PUCS4 exhibited relatively balanced mechanical characteristics with a tensile strength of 26.9 MPa, an elongation at break of 1400%, and toughness of 149.4 MJ m^−3^.

The triple‐dynamic bond polymer network of PUCS was expected to exhibit exceptional mechanical resilience, and subsequently, cyclic tensile tests were conducted to evaluate these properties. The initial step involved conducting a cyclic tensile test on PUCS4, gradually increasing strain to observe its performance changes (Figure 3d). In this continuous cyclic tensile test, there was no waiting period between the consecutive loading cycles. The results demonstrated the excellent elastic properties of the samples, during their initial 10% stretching cycle, the internal hydrogen bonds in the sample rapidly reorganized, enabling the material to quickly return to its original state.^[^ ^28^ ^]^ The 100% strain continuous cycle test (Figure 3e) revealed a substantial hysteresis loop following the initial cycle, indicating significant energy dissipation in the sample. Additionally, noticeable residual strain was observed in the stretching region, resulting in a phenomenon triggered by dynamic fracture. The hysteresis loop of the second cycle was reduced, and the energy dissipation was significantly lower than the first cycle, due to the insufficient reformation of sacrificial bonds within the limited time.^[^ ^29^ ^]^ Furthermore, multiple cycles led to a decrease in hysteresis loops, indicating the continuous breakdown and reassembly process of sacrificial bonds. After the material was placed at room temperature for 2 h, the loading and unloading curves exhibited a remarkable similarity to the initial cycle, demonstrating excellent elastic recovery capability of the prepared elastomer. The samples exhibited consistent trends across different strain rates (Figure S5, Supporting Information) and at higher cyclic strains (300%). Notably, the dumbbell shaped elastomer (0.14 g) demonstrated remarkable mechanical properties and elastic resilience, and it could easily lift objects weighing 2.5 kg (equivalent to 17 000 times its own weight, Figure 3c), with near‐complete recovery after a period of relaxation.

The self‐healing performance of PUCS was systematically evaluated with surface scratch recovery and mechanical property healing tests. The scratches on the surface of the samples were characterized by optical microscopy. As shown in Figure 3g, complete restoration of surface scratches was observed after maintaining the material at 50 °C for 1 min. The damaged scratch samples were subjected to healing for 2 h, and the healing properties of the samples showed an upward trend with increasing Cu^2+^ to HDES molar ratios. The self‐healing efficiency of PUCS4 fracture deformation reached a substantial rate of 97% (elongation), and the tensile strength (23.4 MPa) and toughness (129.6 MJ m^−3^) were effectively restored (Figure 3f and Table S3, Supporting Information). The presence of reversible hydrogen bonding, metal coordination, and disulfide linkages in the polymer network contributed to this phenomenon. Damage to the sample disrupted the reversible bonds in the material, leading to strong interactions between the metal ions and ligands that facilitated recovery at a lower temperature. The breakage and recombination of disulfide bonds could be explained by a radical‐mediated mechanism. Specifically, upon cleavage of a disulfide bond, a sulfur‐centered free radical was generated, which subsequently attacked the other disulfide bonds to facilitate the formation of new bonds.^[^ ^30^ ^]^ This process was also feasible under moderate temperatures. However, with an increasing number of metal coordination bonds, the cross‐linking strength between polymer chains also rapidly increased, impeding the mobility of molecular chains. Moreover, the reduction in disulfide bonds had a detrimental impact segment motion, reducing the presence of reversible bonds. Consequently, an elastic material with a higher proportion of metal coordination bonds and lower proportion of disulfide bonds exhibited enhanced tensile strength while experiencing reduced self‐healing efficiency. To further validate this, samples containing only coordination bonds or solely disulfide bonds were synthesized. As illustrated in Figure S6, Supporting Information, the coordination bond‐exclusive elastomer (PUC) exhibited high mechanical strength but limited self‐healing capacity. In contrast, the disulfide bond‐only elastomer (PUS) demonstrated inferior mechanical properties yet relatively enhanced self‐healing performance. The PUCS4 elastomer, incorporating both disulfide and coordination bonds, achieved balanced mechanical properties alongside optimal self‐healing performance. These results confirm that the synergistic interplay between metal coordination bonds and disulfide bonds effectively enhances the self‐healing capability of elastomers. As shown in Figure 3h and Table S5, Supporting Information, the self‐healing behavior of PUCS4 demonstrated evident temperature dependence. Even when subjected to a healing process at 30 °C for 2 h, the damaged sample still exhibited inadequate tensile strength and elongation at break. However, these issues could be significantly ameliorated by increasing the temperature.

**Figure 4:**
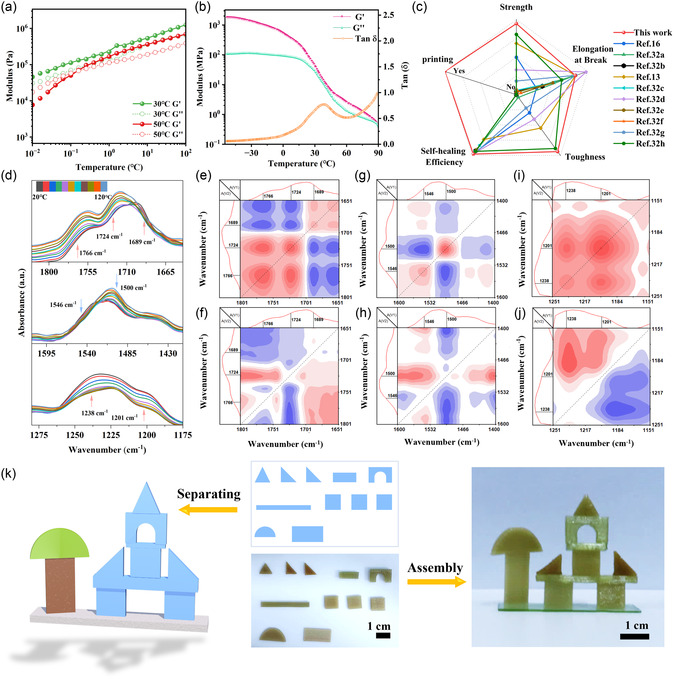
Temperature‐dependent self‐healing behavior and FDM 3D printing demonstration of PUCS4. a) Frequency dependence of G′ and G″ modulus of PUCS4 polymers at 30 and 50 °C. b) DMA curves recorded of PUCS4. c) Comparison of PUCS4 elastomers with other reported polymers based on coordination or disulfide bonds. d) Temperature‐dependent infrared spectra of PUCS4 polymer and corresponding e,g,i) 2D‐COS synchronous spectra and f,h,j) asynchronous spectra. k) Assemble objects with 3D printed parts like building bricks.

The correlation between polymer self‐healing behavior and temperature was further investigated through rheological testing, dynamic mechanical analysis (DMA), and in situ FTIR characterization conducted on PUCS4. The rheological results from frequency scanning (**Figure** **4**a) demonstrated significant separation between the energy storage modulus (G′) and loss modulus (G″) of the sample at 30 °C, indicating pronounced elastic behavior. When the temperature was increased to 50 °C, an intersection between G′ and G″ emerged in the low frequency regions, signifying enhanced polymer chain mobility in the elastomer, the transition of metal coordination bonds from equilibrium to dissociated states,^[^ ^31^ ^]^ reduced mechanical strength, increased polymer mobility, and the metathesis of disulfide bonds.^[7b]^ Consequently, the elastomer experienced softening and transitioned from an elastic state to the viscoelastic state while exhibiting enhanced self‐healing capabilities. The temperature‐dependent DMA results (Figure 4b) demonstrated that PUCS4 exhibited a glass transition temperature (*T*_g_) of ≈39 °C. Moreover, the elastic modulus of PUCS4 experienced a rapid decrease with increasing temperature, indicating the accelerated mobility of polymer chains and the enhanced fracture recombination of reversible bonds. These variations were favorable for achieving higher healing efficiency. The material exhibited distinct advantages compared to recently reported materials prepared using disulfide or metal coordination bonds (Figure 4c).^[^ ^13^, ^16^, ^32^ ^]^

The temperature response mechanism of hydrogen and metal coordination bonds was verified through the in situ temperature changing infrared spectroscopy (Figure S8, Supporting Information) and 2D correlation spectroscopy (2D‐COS). The application of 2D‐COS enhanced initial spectral resolution and provided comprehensive insights into molecular structure and chain dynamics. The variable temperature FTIR spectra between 1175–1820 cm^−1^ revealed the presence of multiple auto‐peaks at 1201, 1238, 1500, 1546, 1689, 1724, and 1766 cm^−1^ throughout the temperature variation (Figure 4d). These peaks were attributed to a variety of molecular vibrations, including coordination bond ν(N—O), ν_free_(N—O),^[^ ^33^ ^]^ medium strength hydrogen bonding interactions ν(N—H), weak hydrogen bonding interactions ν(N—H), ordered hydrogen bonds ν(C=O), and disordered hydrogen bonds ν(C=O) (ester) and ν(C=O), respectively.[^7^, ^34^] The cross‐peaks (Figure 4e–j) observed in the synchronous and asynchronous spectra of PUCS4 corresponded to the vibrational modes at frequencies (1766, 1724), (1724, 1689), (1546, 1500), and (1238, 1201). According to the Noda rule, the sequence relationship of chemical groups experienced changes under external interference, which could be determined by analyzing cross peaks in synchronous and asynchronous spectra.^[^ ^35^ ^]^ If both cross peaks (ν_1_, ν_2_) exhibited positive or negative values, it indicated that ν_1_ changes at a faster rate compared to ν_2_. Conversely, if one peak was positive while the other was negative, it suggested that ν_2_ changed more rapidly than ν_1_. Accordingly, the chemical bonds response speed of PUCS4 during temperature increase followed: 1238 > 1201; 1500 > 1546; and 1766 > 1724 > 1689 cm^−1^. The results indicated the presence of metal coordination interactions and multiple hydrogen bond interactions in PUCS4. During the heating process, a preferential change was observed in the N—H of strong hydrogen and C=O bonds associated with disordered hydrogen bonds, while the free N—O bond coordinated with Cu^2+^ for rapid reversible dissociation association. The results were further supported by the presence of an infrared spectrum peak at 1238 cm^−1^ for Cu^2+^ joining (Figure S2, Supporting Information).^[^ ^33^ ^]^ This demonstrated that increasing the temperature promoted the fracture recombination of reversible bonds in the polymer network and accelerated chain movement, enhancing its self‐healing performance.

The rheological temperature slope results of the PUCS polymer (Figure S9, Supporting Information) indicated a decrease in G′ following an increase in temperature, where the intersection of G′ and G″ appeared below 85 °C. This indicated that the elastomer transitioned from an elastic state to a flowing viscous state, facilitating smooth high‐temperature FDM printing. The thermogravimetric analysis (TGA) results (Figure S10, Supporting Information) demonstrated excellent thermodynamic stability of the sample, as decomposition did not occur until the temperature exceeded 170 °C. The sample will not be damaged during the printing process. The exceptional self‐healing performance of the sample enabled its effective utilization in high‐temperature FDM 3D printing, significantly enhancing interlayer adhesion, mending damage incurred during usage, and prolonging its service life. The assembly of 3D printed small parts resembling building bricks enabled the fabrication of large castle‐like structures (Figure 4k and S9, Supporting Information), eliminating the need for support structures when printing sizable objects. Simultaneously, this also eliminated any potential risks associated with removing support structures. The 3D printing ink could be readily synthesized from affordable commercial chemicals, making it suitable for large‐scale production.

**Figure 5:**
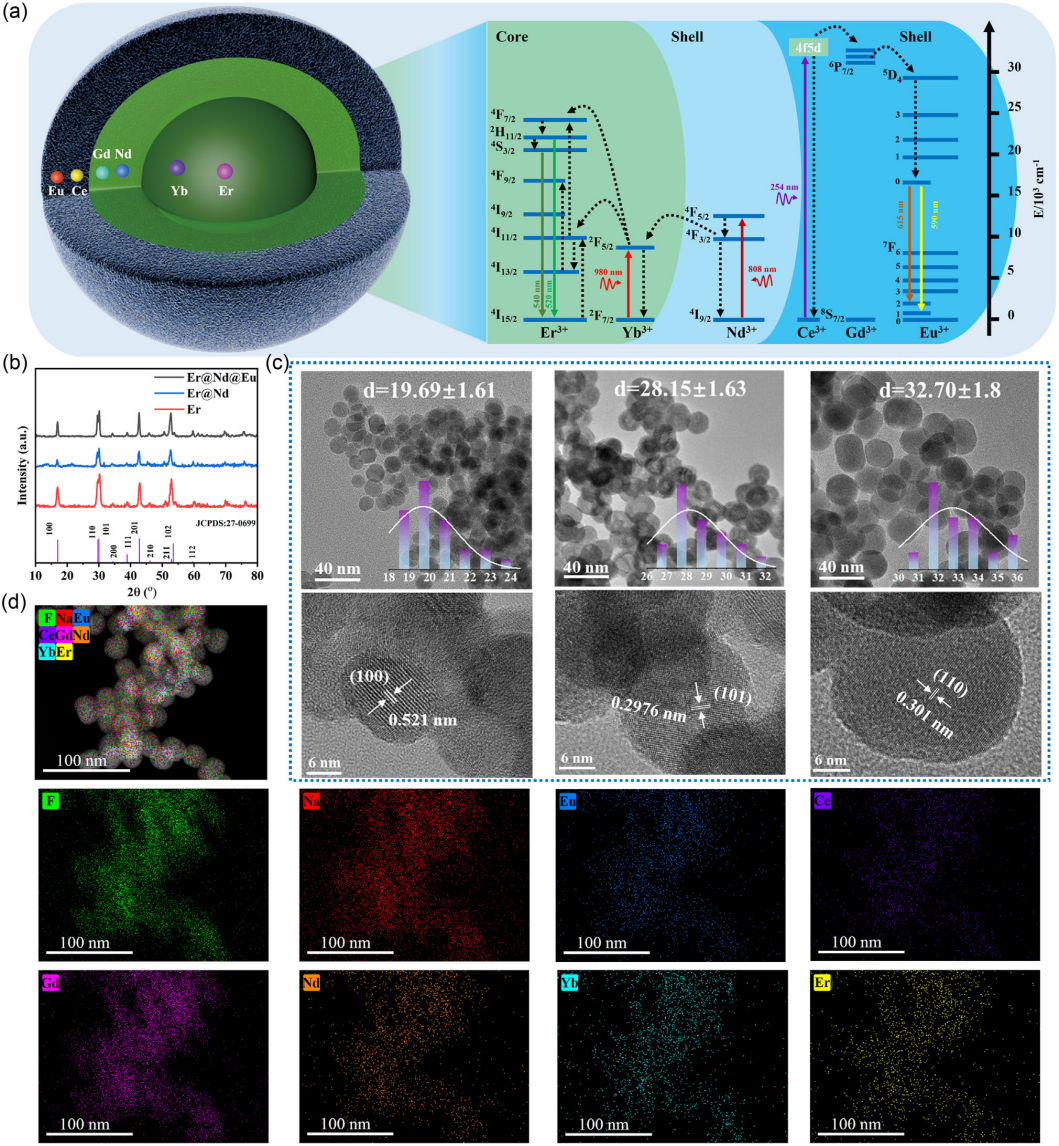
Luminescence mechanism and structural characterization of triple‐mode fluorescent nanoparticles a) Structure and energy transfer mechanism of core–shell fluorescent nanoparticles. b) XRD diffraction patterns of core–shell fluorescent nanoparticles, including Er, Er@Nd, and Er@Nd@Eu. c) TEM and HRTEM images of Er (left), Er@Nd (middle), and Er@Nd@Eu (right) nanoparticles. d) HAADF image and the corresponding EDS mapping for the Er@Nd@Eu nanoparticles.

**Figure 6:**
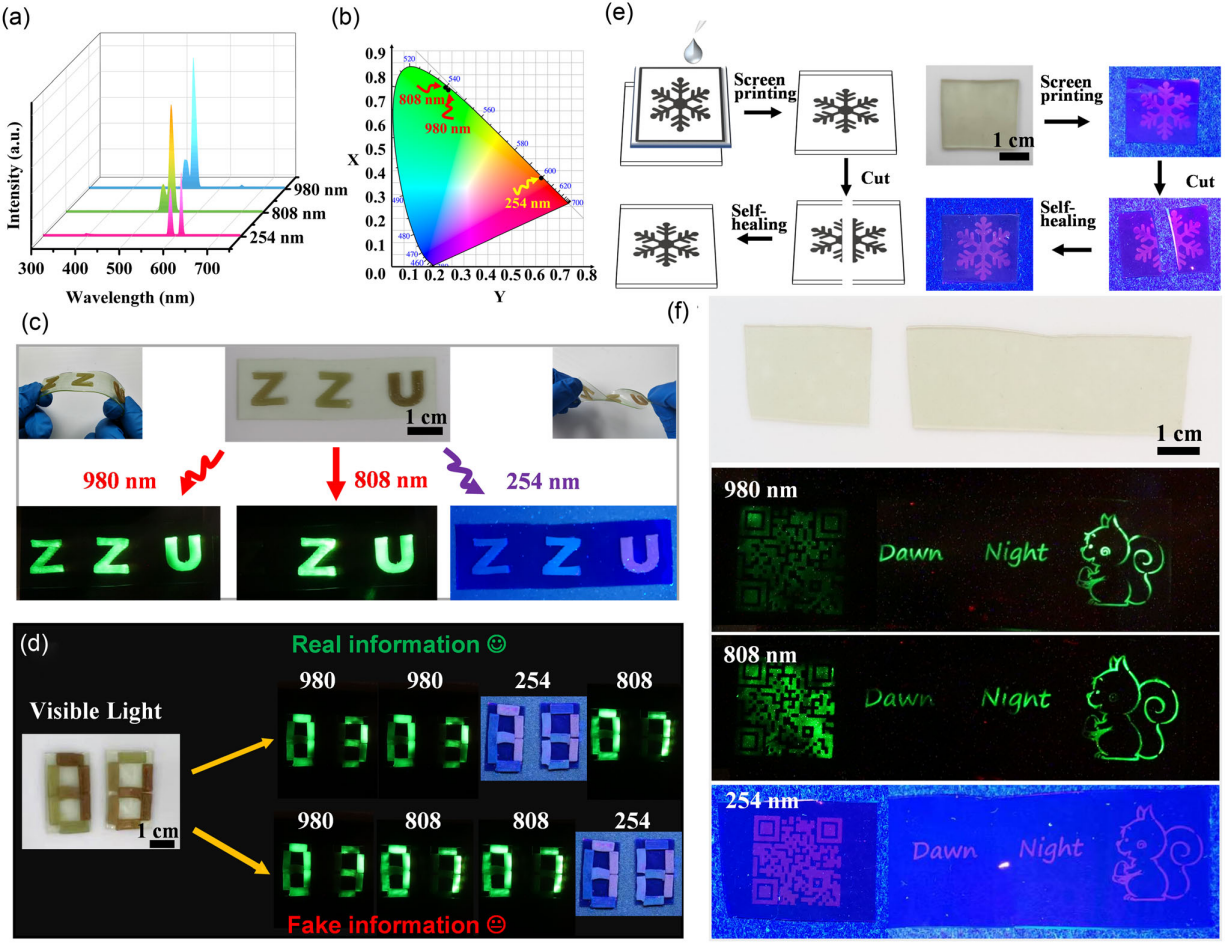
Application of self‐healing optics and fluorescent ink based on PUCS4. a) Fluorescence spectra of DMF dispersion of triple‐mode fluorescent nanoparticles. b) CIE coordinates of triple‐mode fluorescent nanoparticles. c) 3D printed the letter “ZZU” devices by using the fluorescent elastomer incorporated different fluorescent particles. d) 3D printed the cuboid assembly fluorescent coding device and demonstrated its information encryption application. e) Optical devices prepared by screen printing and self‐healing the damaged fluorescent devices. f) Optical device under natural light, 980, 808, and 254 nm excitation, respectively.

The printability of the polymer, along with its excellent healing properties, mechanical properties, and high transparency were used to incorporate up/downconversion fluorescent nanoparticles with significant optical properties into the material. Subsequently, a series of 3D printable fluorescent elastomers and triple‐mode fluorescent inks were successfully prepared. Synthesis of the core–shell fluorescent nanoparticles was achieved by a three‐step coprecipitation method. Initially, core material NaGdF_4_ doped with Er^3+^ and Yb^3+^ was synthesized, followed by the subsequent coating of its shell with NaGdF_4_:Nd. Finally, triple‐mode fluorescent nanoparticles were obtained through the synthesis of NaGdF_4_:Ce and Eu as the outermost layer. The structure of the nanoparticle and energy‐level transition diagram of the luminescence process are illustrated in **Figure** **5**a. The Yb^3+^, Nd^3+^, and Ce^3+^ ions were selected as sensitizing agents to selectively absorb excitation photons at wavelengths of 980, 808, and 254 nm, respectively. The hexagonal phase NaGdF_4_ served as the core host lattice, which facilitated efficient energy transfer from Ce^3+^ to Eu^3+^.^[^ ^36^ ^]^ After the absorption of 808 nm photons, the Nd^3+^ ions were excited to the ^4^F_5/2_ level and subsequently transferred their energy to Yb^3+^, inducing its transition to the ^2^F_5/2_ level. Additionally, Yb^3+^ directly absorbed energy from a photon with a wavelength of 980 nm, facilitating its transition to the ground state, while also transferring the absorbed energy to Er^3+^ ions. The Er^3+^ ions efficiently assimilated the received energy and were excited to the ^4^F_7/2_ level, resulting in the emission of vibrant green fluorescence after returning to the ground state. Under laser irradiation at a wavelength of 254 nm, the Ce^3+^ sensitizers absorbed energy and transferred to the ^5^D_1_ level. The energy was further transferred through Gd^3+^ ions to Eu^3+^, promoting its transition into the excited state (^5^D_4_), which finally emitted red fluorescence after relaxation back to its ground state. Figure S14, Supporting Information, provides a comprehensive list of formulas for energy level transitions. The observed peaks and intensities in the XRD patterns (Figure 5b) of the fluorescent nanoparticles were consistent with the JCPDS standard card (27‐0699) for the hexagonal NaGdF_4_ phase, indicating no change in the crystal structure of the coated rare earth ions. Figure 5c presents the transmission electron microscopy (TEM) structure of the nanoparticles, revealing their uniform spherical and ellipsoid morphology. Following the coating process, the size of the nanoparticles increased from ≈20 to 33 nm. The high‐resolution TEM images revealed distinct lattice fringe spacings of 0.521, 0.2976, and 0.301 nm corresponding to the (100), (101), and (110) lattice planes of the hexagonal phase NaGdF_4_, respectively. X‐ray energy‐dispersive analysis (EDS) demonstrated the spatial distribution of F, Na, Eu, Ce, Gd, Nd, Yb, and Er in the nanoparticles (Figure 5d), with the fluorescence spectra of triple‐mode fluorescent nanoparticles shown in **Figure** **6**a. Red emission peaks were observed at wavelengths of 590 and 615 nm, along with green emission peaks at wavelengths of 520 and 540 nm, respectively. Additionally, the CIE chromaticity coordinates (Figure 6b and Table S6, Supporting Information) for fluorescence emission under excitation wavelengths of 254, 808, and 980 nm were determined as (0.621, 0.376), (0.227, 0.746), and (0.233, 0.742), correspondingly. Notably, bright fluorescent emission from the cyclohexane dispersion of nanoparticles was visible, as shown in Figure S15, Supporting Information. The relationship between the primary emission peak intensity and laser power was fitted using a double logarithm model, and the number of n‐photons involved in the up‐conversion luminescence process was calculated (Figure S14, Supporting Information). The results indicated that the 520 and 540 nm luminescence exhibited a two‐photon mechanism.

The polymer was dissolved in ethyl acetate (ETAC), followed by the dispersion of Er, Er@Nd, and Er@Nd@Eu core–shell fluorescent nanoparticles in the polymer matrix. Subsequently, the compound was added to a polytetrafluoroethylene mold at 65 °C to obtain various fluorescent elastomer films. The letters ZZU were fabricated using 3D printing technology (Figure 6c), and their inherent self‐healing properties allowed them to grow directly on the PUCS base without sustaining damage during bending or twisting. Under 980 nm laser irradiation, ZZU exhibited intense green luminescence. When exclusively exposed to 808 nm irradiation, only light by ZU was emitted, whereas under exclusive exposure to 254 nm irradiation, only U exhibited emission. Therefore, the alteration of irradiation order yielded distinct information. For example, by subjecting the sample to 808 nm laser irradiation twice, followed by one round of 980 nm laser irradiation, and concluding with two rounds of 254 nm laser irradiation, the letter group “ZUZUZZUUU” was obtained, achieving information encryption. The feasibility of this strategy was further validated by 3D printing and assembling a 1 × 0.5 cm cuboid into the pattern shown in Figure 6d. No discernible information was observed without excitation, while under irradiation at wavelengths of 980, 808, and 254 nm, the respective numbers 03, 07, and 71 were displayed (Figure S16, Supporting Information). The configuration of the assembled cuboid could be modified to display various numbers under different excitations. The sequence and frequency of irradiation could be varied to obtain diverse encrypted digital codes. For example, by subjecting it to two rounds of 980 nm irradiation followed by one round each of 254 and 808 nm irradiation resulted in an encrypted digital password: 03037107. By contrast, changing the number or order of irradiations could lead to the appearance of error messages (Figure 6d). The high level of customization, user‐friendly operation, and rapid manufacturing capabilities of 3D printing facilitated the rapid and efficient customization of various luminous patterns or codes to meet encryption requirements. A fluorescent ink was also formulated by dissolving polymers with Er@Nd@Eu core–shell fluorescent nanoparticles into a DMF solution. Following the screen‐printing technique (Figure 6e), an imperceptible 2D pattern with high resolution was accurately replicated, as shown in Figure 6f. The prepared fluorescent design remained invisible under visible light. Notably, upon exposure to 980, 808, and 254 nm irradiation, the design emitted vibrant fluorescence that unveiled legible information. Furthermore, the self‐healing performance of the elastomers facilitated the restoration of severed fluorescence devices (Figure 6e). The aforementioned applications effectively utilized the immense potential of self‐healing fluorescent devices in the fields of information encoding, encryption, and fluorescence‐based anticounterfeiting. The favorable mechanical properties and self‐healing capabilities of PUCS elastomers effectively address the wear and damage issues of anticounterfeiting devices during use, while the triple‐mode luminescence enhances information encryption security. Additionally, 3D printing significantly simplifies the preparation of fluorescent devices with patterns. The aforementioned applications based on the PUCS elastomers demonstrate significant application potential in the fields of information encoding, encryption, and fluorescence‐based anticounterfeiting.

## 3 Conclusion

This study successfully demonstrated the design and synthesis of a 2D/3D printable self‐healing PUCS elastomer, followed by the fabrication of self‐healing fluorescence devices based on the elastomer. The elastomer's metal coordination, hydrogen, and disulfide bonds contributed to its exceptional mechanical characteristics, comprising a high tensile strength (26.9 MPa), elongation at break (1400%), toughness (149.4 MJ m^−3^), and self‐healing ability (97%). The elastomer could be used in high‐temperature FDM 3D printing, and due to its exceptional self‐healing efficiency, it effectively addressed the limitations associated with FDM 3D printing, such as inadequate interlayer adhesion and the need for support structures. Finally, the elastomer doped with triple‐mode core–shell fluorescent nanoparticles could emit strong fluorescence when exposed to irradiation at 980, 808, and 254 nm. 2D/3D printing enabled the rapid customization of various fluorescence devices, facilitating encryption through coding for information security, allowing for the effective concealment of fluorescence information, and supporting self‐healing capabilities after damage. These features demonstrated significant potential in the fields of information coding and intelligent anti‐counterfeiting.

## 4 Experimental Section

4.1

4.1.1

##### Materials

PTMEG (*M*_n_ ≈ 1000), IPDI (mixture of isomers) (99%), ditin butyl dilaurate (DBTDL, 95%), DMG (98%), HDES (≥90%), ETAC (99.5%), copper(II) chloride (CuCl_2_, 98%), DMF (99.5%), gadolinium chloride hexahydrate (99.9%), ytterbium(III) chloride hexahydrate (99.9%), erbium chloride hexahydrate (99.995%), europium chloride hexahydrate (99.99%), neodymium(III) chloride hexahydrate (99.9%), oleic acid (85%), and 1‐Octadecene (>90%) were purchased from Aladdin Chemistry Co., Ltd. Cerium chloride hexahydrate (99.99% metals basis) and glycerol (≥99%) were obtained from Macklin Inc. Ammonium fluoride (AR, 98%) and sodium hydroxide (≥98%) were received from Shanghai YiEn Chemical Technology Co., Ltd. Acetone (≥99.5%) and methanol (≥99.5%) were bought from Sinopharm Chemical Reagent Co., Ltd. All reagents were utilized in their as‐received state without additional purification.

##### Synthesis of Transparent PUCS Polymers

The synthesis pathway of PUCS is as follows. PUCS polymers with different content of hard and soft segments were synthesized by the same method. The detailed dosage of experimental was listed in Table S1, Supporting Information. Typically, the PTMEG was placed into a three‐necked glass flask and subjected to stirring for a duration of 2 h under an argon atmosphere at 120 °C. After cooling to 70 °C, IPDI (6.03 mL), DBTDL (40 μL), and ETAC (10 mL) were sequentially added with stirring for 1 h. When the temperature lowered to 40 °C, HDES (0.735 mL) and ETAC (10 mL) were injected in the prepolymer with continuous stirring. Subsequently, DMG (1.18 g) was dispersed in ethyl acetate (ETAC), while CuCl_2_ (0.011 g) and glycerol (0.223 g) were dissolved in acetone. Both solutions were then added to the prepolymer and stirred for 90 min. The mucus‐like reaction was poured into a polytetrafluoroethylene mold and subjected to vacuum deaeration before further curing in an oven to produce the PUCS4.

##### Preparation of Triple‐Mode Core@shell Fluorescent Nanoparticles

The process of preparing NaGdF_4_:Yb, Er@NaGdF_4_:Nd@NaGdF_4_:Ce, Eu by high temperature coprecipitation is as follows. The precursors, including a mixture of OA/ODE (7.5:15 mL) and 2 mmol of ReCl_3_ (Gd:Yb:Er = 80:18:2), were added into a three‐necked glass bottle. The suspension was stirred at 160 °C for 30 min with argon flowing to facilitate dissolution. The reaction was allowed to cool down naturally to ambient temperature; the methanol (27 mL) solution of NaOH (200 mg) and NH_4_F (296 mg) was quickly mixed and poured into the above mixture, and stirred at 48 °C for half an hour. Subsequently, the mixture was kept at 90 °C for 20 min to remove residual methanol. The solution was subjected to a temperature of 300 °C, stirred for another 90 min, and then cooled to ambient temperature. The obtained mixture was centrifugated with ethanol and cyclohexane and collected, resulting in the isolation of core nanoparticles (designated as Er). The shell layer of NaGdF_4_:Nd (1 mmol, Gd:Nd = 80:20) was prepared by the same method to coat the core Er but with half the amount of all materials. Before the introduction of the methanol solution of NaOH/NH_4_F, the cyclohexane solution containing Er nanoparticles was added into the solution. The core–shell nanoparticles obtained were labeled as Er@Nd. In the same way, a second layer of 1 mmol NaGdF_4_:Ce, Eu (Gd:Ce:Eu = 70:15:15) was wrapped over Er@Nd to obtain Er@Nd@Eu.

##### Synthesis of Fluorescent Self‐Healing Materials

Typically, 5 g of PUCS sample and 50 mL of ETAC were introduced into a closed flask, which was subsequently put in a 60 °C oil bath and stirred until the sample achieves complete dissolution. Afterward, the core–shell fluorescent nanoparticles were dispersed in cyclohexane and subjected to stirring for a duration of one hour. The resulting solution obtained was transferred into a Teflon mold and cured in an oven. The film was dissolved in DMF to prepare fluorescent ink for screen printing.

##### Characterizations

The transmittance of PUCS elastomer was tested by a UV3600i plus spectrophotometer (Shimadzu, Japan). The FTIR spectra of elastomers were characterized using a TENSOR II (Bruker) spectrometer range from 4000 to 650 cm^−1^ featuring a resolution of 1.5 cm^−1^. The XRD pattern was characterized with Rigaku SmartLab SE at a scanning speed of 10° min^−1^. Raman spectra in the range of 350−750 cm^−1^ were recorded on a LabRAM Soleil Raman Microscope (Horiba) under 532 nm irradiation laser. The XPS was performed by a Scientific K‐Alpha (Thermo, American). Molecular weight (*M*_n_ and *M*_w_) and PDI were carried out by a GPC instrument (Agilent, America), using DMF as the mobile phase; 5 mg of the sample was dissolved in 1 mL of solvent. The analysis was performed under a flow rate of 1 mL min^−1^ with an injection volume of 100 μL. DSC was evaluated in a DSC−60 Plus (Shimadzu) ranging from −70 to 150 °C at a heating rate of 10 °C min^−1^ in a N_2_ atmosphere. TGA test was measured using a TGA‐50 (Shimadzu) across 30 to 700 °C with a heating speed of 10 °C min^−1^ under N_2_ atmosphere. The microphase separation morphology of PUCS self‐healing elastomers was performed by an AFM noncontact mode (Bruker nanowizard 4XP, Germany). The stress–strain curves were recorded from tests conducted on dumbbell‐shaped samples using a ZQ‐990LB tester equipped with a 200 N load cell and a strain rate of 100 mm min^−1^. The dynamic thermomechanical properties (modulus and tan *δ*) of the PUCS were tested using temperature scanning from −100 to 60 °C in the tensile mode of the DMA TA Q800 instrument (Waters, America) with heating rate of 3 °C min^−1^. The TA HR10 rheometer (Waters, America) performed frequency scans at various temperatures, utilizing a strain of 0.1% and a frequency between 0.01 and 100 rad s^−1^. Additionally, temperature ramp tests were recorded from 30 to 120 °C with a heating rate of 3 °C min^−1^, maintaining a frequency of 1 Hz. The mechanical self‐healing performances of PUCS are demonstrated through scratches, subsequent healing at various temperatures and times, followed by tensile tests. The scratch self‐healing property was observed under a polarized optical microscope (BM‐62XCD) and the temperature was controlled with a hot station (TES120T‐PM). The in situ temperature‐dependent FTIR spectra were recorded with an IRAffinity‐1S spectrometer (Shimadzu) with a wavelength range of 4000−600 cm^−1^, a temperature range of 20−120 °C, and an interval temperature of 10 °C min^−1^. The obtained data were subjected to 2D correlation spectroscopy analysis. The 3D printing process was conducted using a Bio‐ArchitectSR (Regenovo, China) printer equipped with a nozzle diameter of 0.2 mm. The PUCS sample was cut into small pieces and added it to the barrel. The 3D printing temperature is 150 °C, the nozzle diameter is 0.2 mm, and the extrusion speed and printing speed are 0.05 and 6 mm s^−1^, respectively. The TEM pictures and EDS mapping of fluorescence nanocrystals were determined by JEOL JEM‐2100F and Oxford Instruments X‐Max 80T. The fluorescence spectroscopy was acquired utilizing a Techcomp FL‐970 Plus spectrometer outfitted with 980 and 808 nm lasers. Canon EOS 850D camera was used to capture fluorescent images.

## Conflict of Interest

The authors declare no conflict of interest.

## Supporting information

Supplementary Material

## Data Availability

The data that support the findings of this study are available from the corresponding author upon reasonable request.
