# Supplementary material for: Fluorescent Self‐Healing Elastomers with Triple Dynamic Bonds for 2D/3D Printed Information Encryption
Source: Small Sci. 2025 Jul 30;5(9):2500091. doi: 10.1002/smsc.202500091 (PMC12412527; doi:10.1002/smsc.202500091)
Supplement: Supplementary file 1 — Supplementary Material [file SMSC-5-2500091-s001.pdf]

Supporting Information

Fluorescent Self-Healing Elastomers with Triple Dynamic Bonds for 2D/3D Printed Information Encryption

*Dai Yang[#], Qingyong Tian[#], Jingyang Li, Xiaoqing Sui. Shuiren Liu, Xiaoguang Hu, Qingqing Sun, Linlin Zhang, Mingjun Niu, Xuying Liu, Weijing Yao**

School of Materials Science and Engineering, Henan Institute of Advanced Technology, Zhengzhou University, Zhengzhou 450001, P. R. China.

[#] These authors contributed equally.

Table S1 Specific synthetic parameters of PUCS polymer and corresponding molar ratio.

Sample	PTMEG (*Mn*, g)	IPDI (g)	DMG (g)	$CuCl_2$ (g)	HDES (g)	DBTDL (g)	Glycerol (g)	Molar Ratio (PTMEG: IPDI:DMG: HDES)
PUCS1	1000, 10 g	6.41	1.8819	0.0174	0.00	0.042	0.223	1:2.88:1.62:0.0
PUCS2	1000, 10 g	6.41	1.6497	0.0153	0.308	0.042	0.223	1:2.88:1.42:0.2
PUCS3	1000, 10 g	6.41	1.4174	0.0131	0.617	0.042	0.223	1:2.88:1.22:0.4
PUCS4	1000, 10 g	6.41	1.1852	0.0110	0.925	0.042	0.223	1:2.88:1.02:0.6
PUCS5	1000, 10 g	6.41	0.9529	0.0088	1.234	0.042	0.223	1:2.88:0.82:0.8
PUS	1000, 10 g	6.41	1.1852	0	0.925	0.042	0.223	1:2.88:1.02:0.6
PUC	1000, 10 g	6.41	1.1852	0.0110	0	0.042	0.223	1:2.88:1.02:0.0
PUCCS	1000, 10 g	6.41	1.1852	0.0352	0.925	0.042	0.223	1:2.88:1.02:0.6

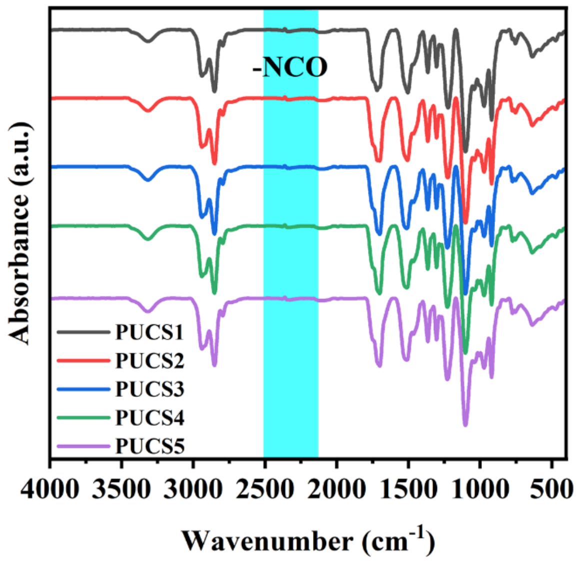

Figure S1 FTIR spectra of different PUCS polymers.

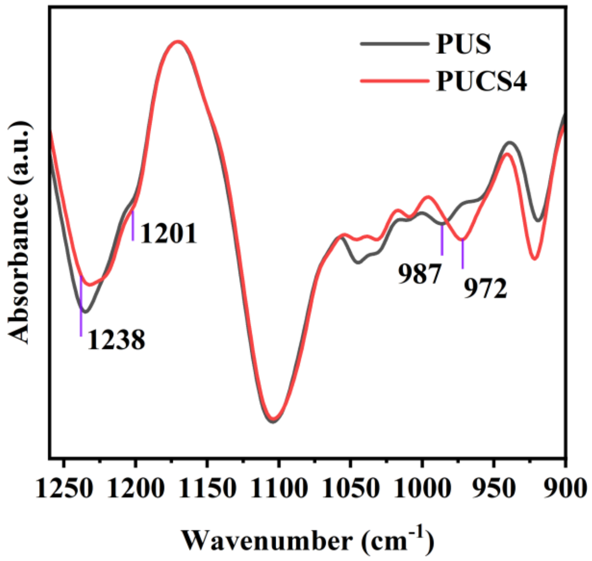

Figure S2 FTIR spectra of PUS and PUCS polymers.

Table S2 Molecular weight and mechanical properties of PUCS self-healing elastomers with different preparation parameters.

Sample	Mn (g/mol)	Mw (g/mol)	PDI	Tensile Strength (MPa)	Elongation at Break (100%)	Toughness (MJ/m^3)
PUCS1	36810	66273	1.80	46.6±0.6	10.0±0.1	233.6±5.5
PUCS2	37649	70342	1.87	38.3±1.0	12.7±0.6	215.4±13.2
PUCS3	38180	70032	1.83	30.2±0.8	13.1±0.5	157.1±8.9
PUCS4	41992	79054	1.88	26.9±2.8	14.0±0.5	149.4±19.7
PUCS5	37827	71505	1.89	9.3±0.7	20.2±1.0	70.9±9.6

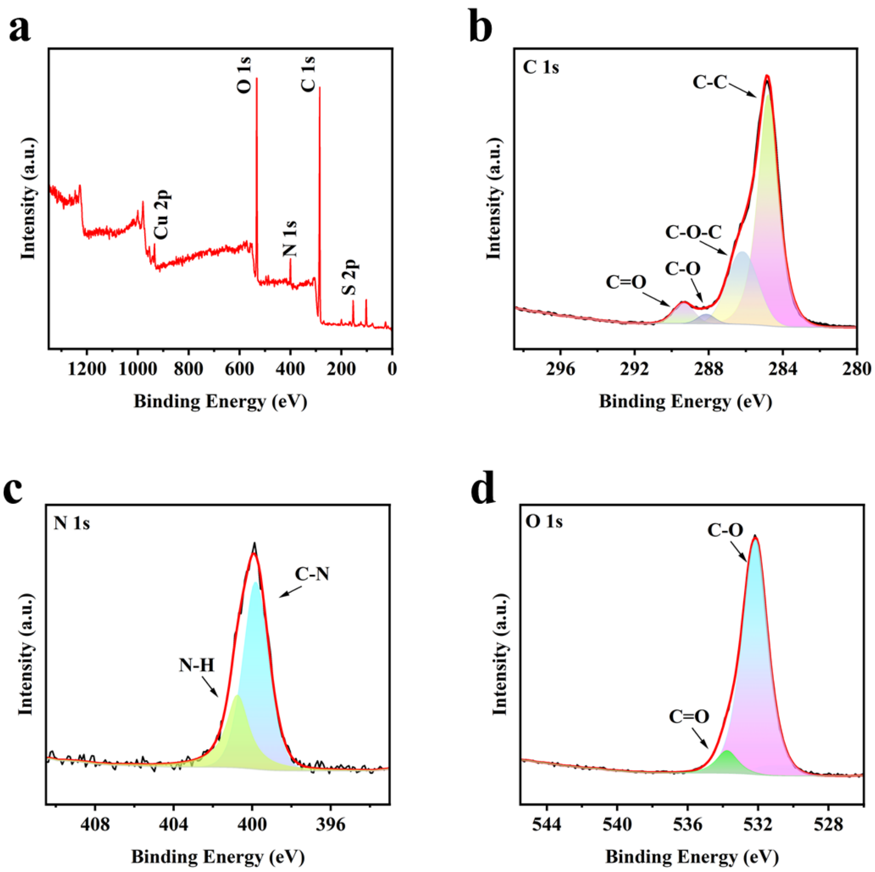

Figure S3 Full XPS spectrum and C 1s, N 1s and O1s of the PUCCS polymer.

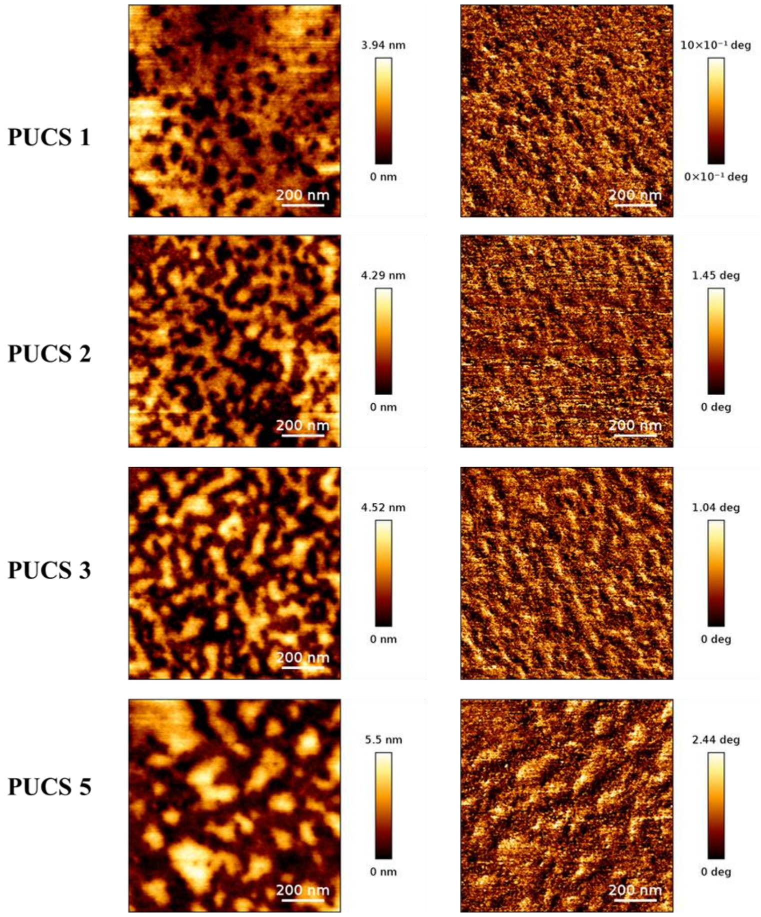

Figure S4 AFM images of PUCS elastomers, the height images (left) and phase images (right).

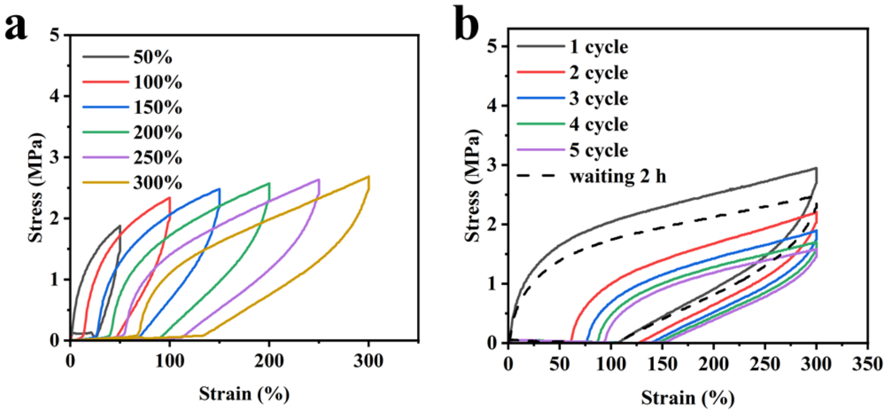

Figure S5 (a) Sequential cyclic tensile curves of PUCS at different strains without waiting time between two consecutive loadings. (b) The cyclic loading-unloading curves of PUCS with a strain of 300%. There was no waiting time between two consecutive cyclic tensile (cycle 1-cycle 5).

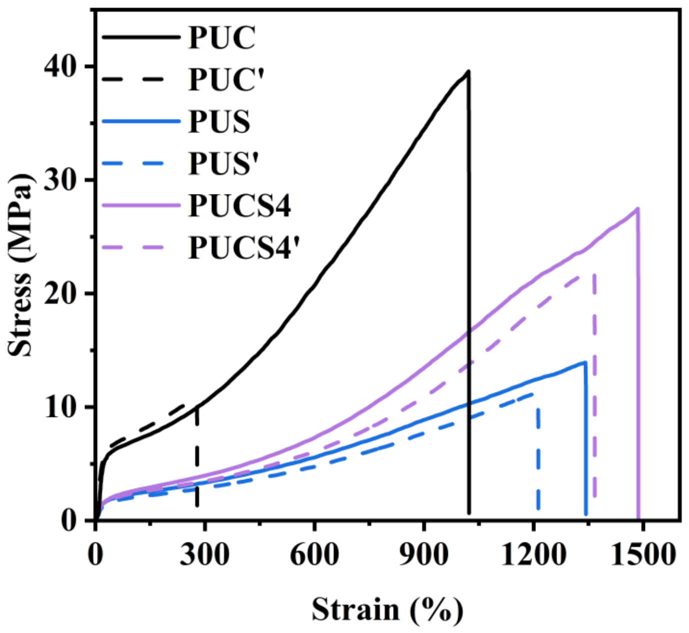

Figure S6 Stress strain curves and self-healing curves (2 h at 50°C) of different elastomers.

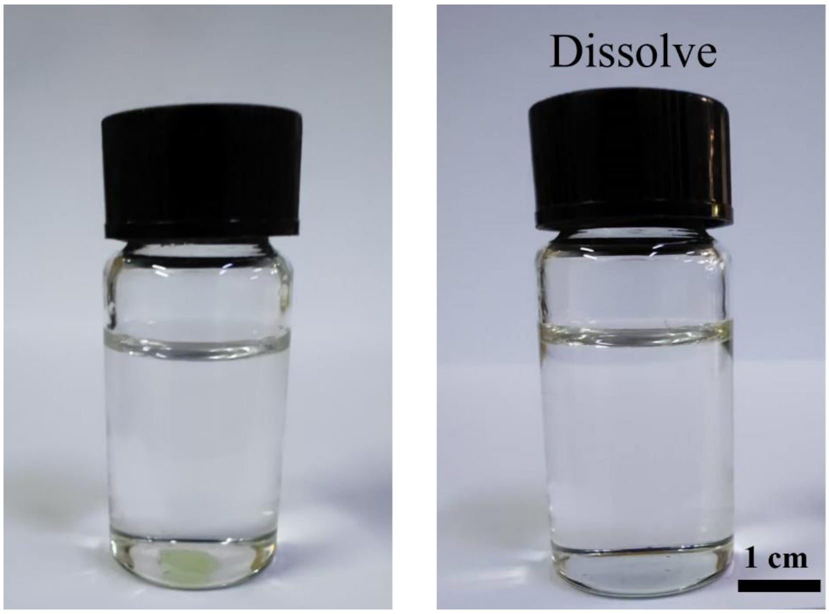

Figure S7 Dissolve 20 mg of sample in 4 mL of DMF solution. (20 mg of the sample was immersed in 4 mL of solvent, corresponding to a concentration of 5 mg/mL required for GPC testing. Complete dissolution was achieved within approximately 12 hours.)

Table S3 Self-healing efficiency of different PUCS elastomers.

Sample	Intact			Healed 50℃ 2 h		
	Tensile Strength (MPa)	Elongation at Break (100%)	Toughness (MJ/m^3)	Tensile Strength (MPa)	Elongation at Break (100%)	Toughness (MJ/m^3)
PUCS1	46.6±0.6	10.0±0.1	233.6±5.5	27.2±2.4	6.07±0.4	89.9±10.6
PUCS2	38.3±1.0	12.7±0.6	215.4±13.2	26.3±2.3	10.5±1.0	130.9±24.5
PUCS3	30.2±0.8	13.1±0.5	157.1±8.9	23.8±1.4	12.4±1.1	123.8±22.3
PUCS4	26.9±2.8	14.0±0.5	149.4±19.7	23.4±2.8	13.6±0.4	129.6±22.5
PUCS5	9.3±0.7	20.2±1.0	70.9±9.6	8.2±0.3	17.3±1.0	54.4±2.9

Table S4 Self-healing efficiency of different elastomers.

Sample	Intact			Healed 50℃ 2 h		
	Tensile Strength (MPa)	Elongation at Break (100%)	Toughness (MJ/m^3)	Tensile Strength (MPa)	Elongation at Break (100%)	Toughness (MJ/m^3)
PUC	39.1±1.2	10.0±0.3	191.5±13.4	11.1±2.1	2.8±0.5	23.5±7.1
PUS	13.6±1.0	13.2±0.6	87.9±10.4	10.8±1.3	11.8±0.6	62.5±10.9
PUCS4	26.9±2.8	14.0±0.5	149.4±19.7	23.4±2.8	13.6±0.4	129.6±22.5

Table S5 Mechanical self-heling properties for PUCS4 polymers under diverse healing conditions.

Self-healed Sample	Tensile Strength (MPa)	Elongation at Break (100%)	Toughness (MJ/m^3)
Intact	26.9±2.8	14.0±0.5	149.4±19.7
30℃-2 h	2.8±0.4	2.6±1.0	5.6±2.6
40℃-2 h	19.8±1.7	11.0±0.9	88.3±11.3
50℃-2 h	23.4±2.8	13.6±0.4	129.6±22.5

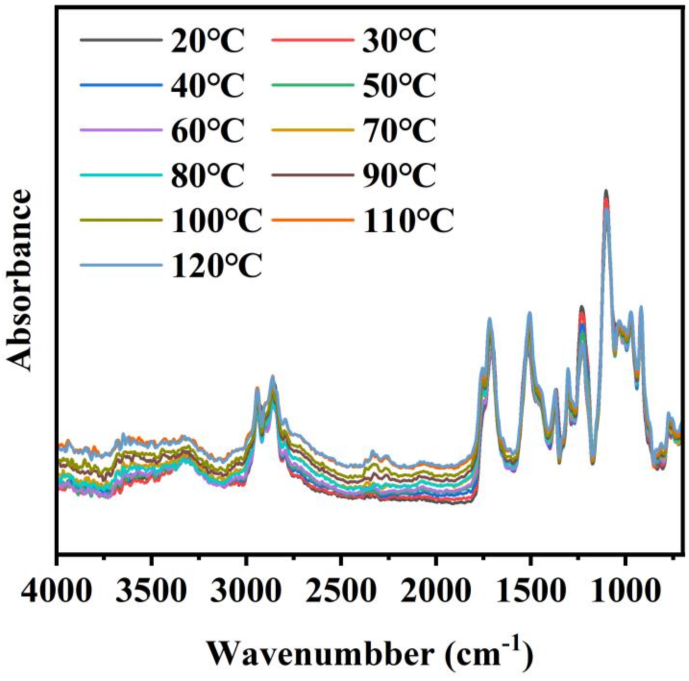

Figure S8 Temperature dependent infrared spectra of PUCS4 polymer.

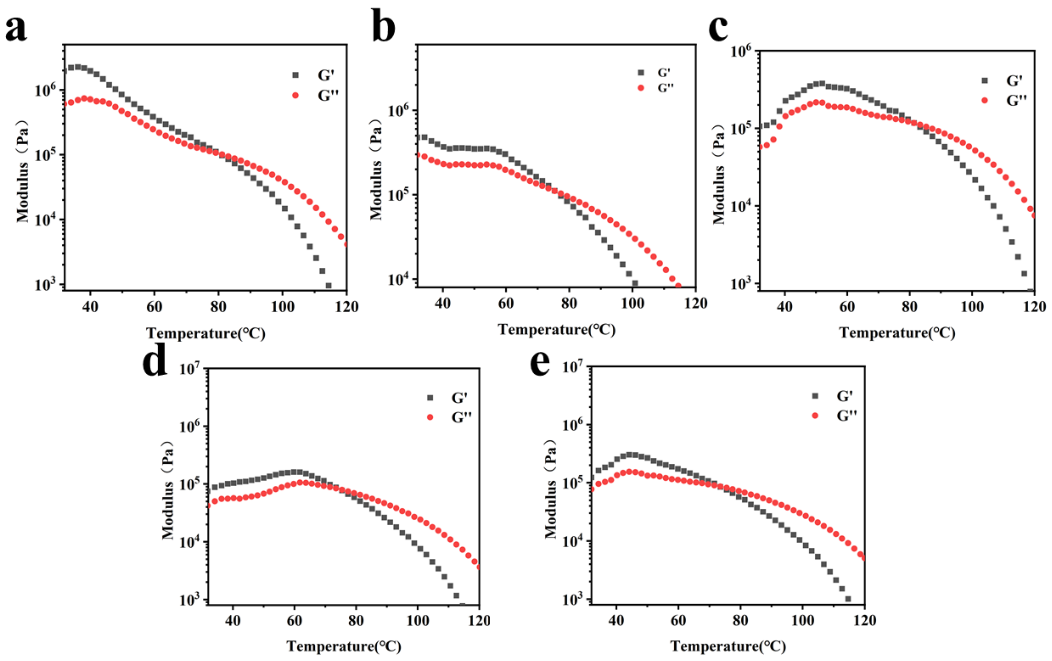

Figure S9 Rheological diagram of PUCS elastomers energy storage modulus and loss modulus changing with temperature. (a) PUCS1. (b) PUCS2. (c) PUCS3. (d) PUCS4. (d) PUCS5.

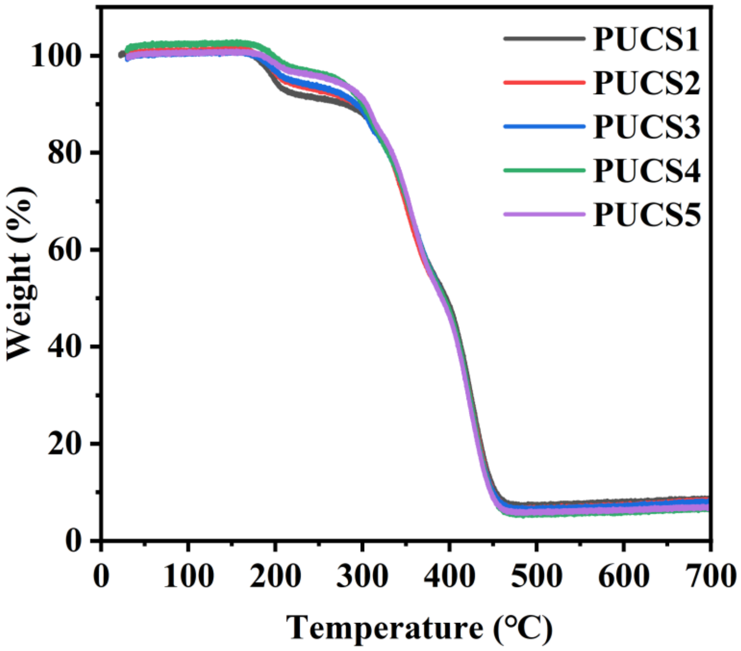

Figure S10 TGA curves recorded of PUCS polymers.

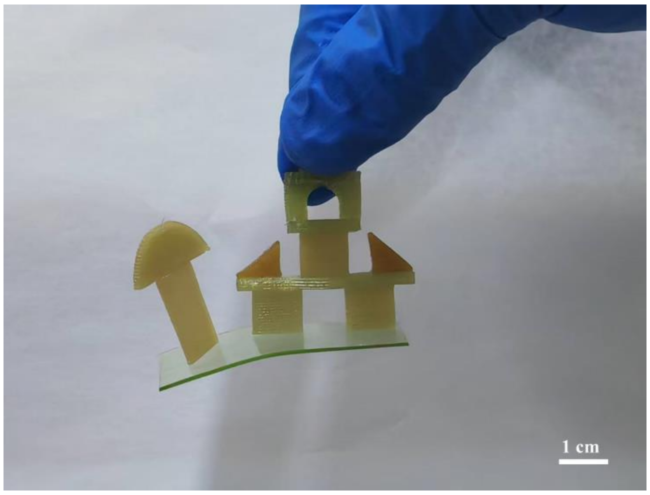

Figure S11 The 3D printed components are assembled and grown together.

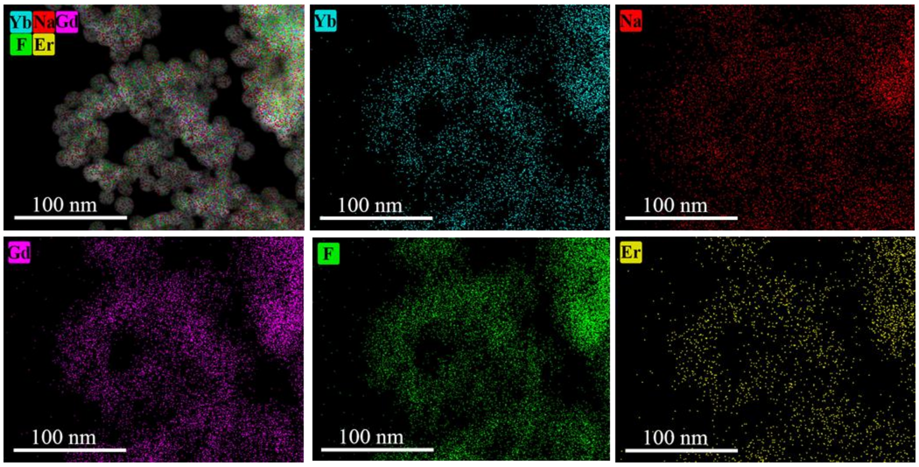

Figure S12 EDS mapping and element distribution for the as-synthesized Er nanoparticles.

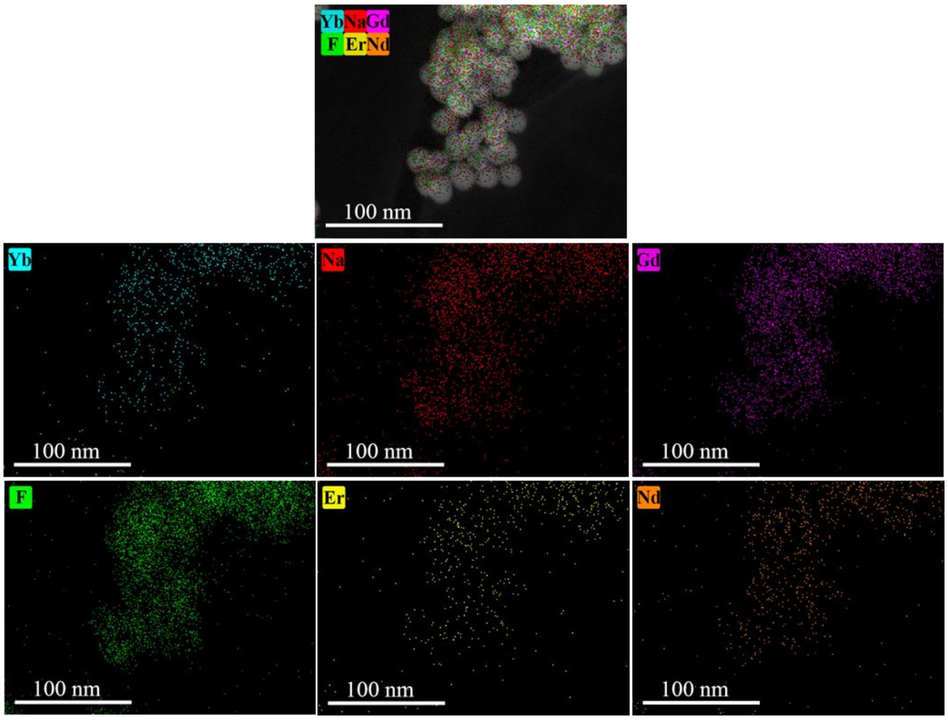

Figure S13 EDS mapping and element distribution for the as-synthesized Er@Nd nanoparticles.

Table S6 CIE coordinates of core-shell fluorescent nanoparticles.

Excitation	Emission colors of CIE color coordinates	
	X	Y
**254 nm**	0.621	0.376
**808 nm**	0.227	0.746
**980 nm**	0.233	0.742

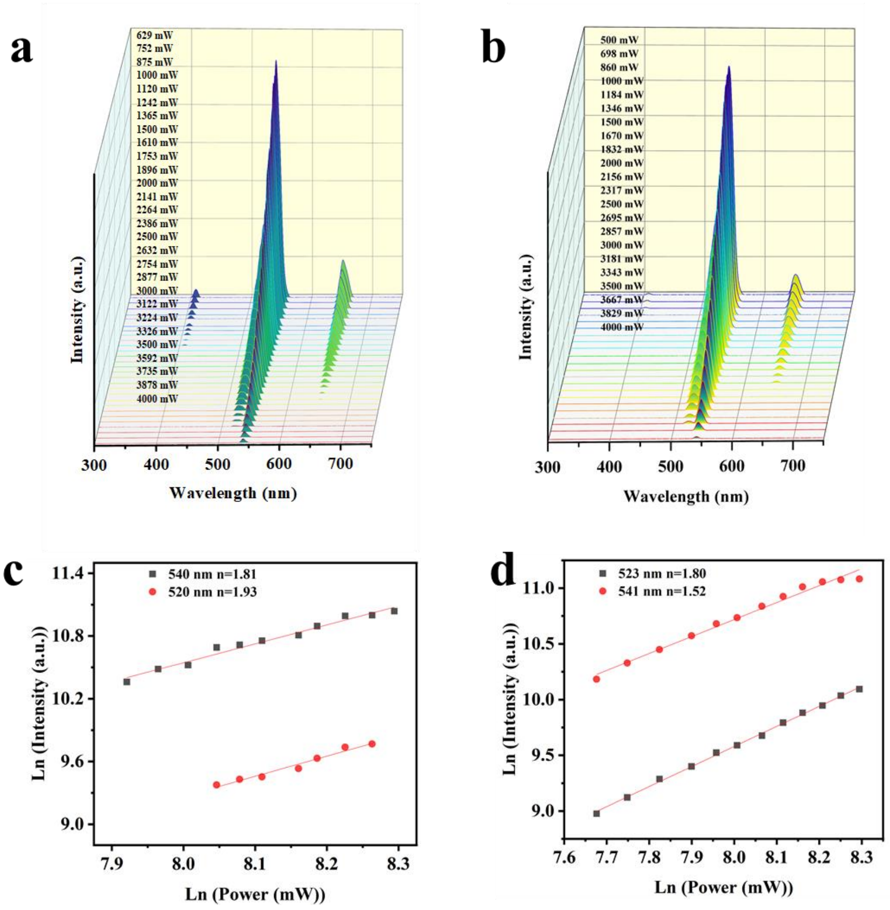

Figure S14 Fluorescence spectra as functions of laser power for core-shell fluorescent nanoparticles under the excitation of (a) 980 nm and (b) 808 nm, and (c, d) the corresponding n-photon processes.

The energy transition formulas:

$$ {}^{2}F_{7/2}(Yb^{3+}) + hv(980\,nm) \xrightarrow{GSA} {}^{2}F_{5/2}(Yb^{3+}) \qquad \text{(S1-1)} $$

$$ {}^{4}I_{9/2}(Nd^{3+}) + hv(808\,nm) \xrightarrow{GSA} {}^{4}F_{5/2}(Nd^{3+}) \qquad \text{(S1-2)} $$

$$ {}^{4}F_{5/2}(Nd^{3+}) \rightarrow {}^{4}F_{3/2}(Nd^{3+}) + {}^{2}F_{7/2}(Yb^{3+}) \xrightarrow{ET1} {}^{2}F_{5/2}(Yb^{3+}) \qquad \text{(S1-3)} $$

$$ {}^{2}F_{5/2}(Yb^{3+}) + {}^{4}I_{15/2}(Er^{3+}) \xrightarrow{ET2} {}^{2}F_{7/2}(Yb^{3+}) + {}^{4}I_{11/2}(Er^{3+}) \qquad \text{(S1-4)} $$

$$ {}^{2}F_{5/2}(Yb^{3+}) + {}^{4}I_{15/2}(Er^{3+}) \xrightarrow{ET3} {}^{2}F_{7/2}(Yb^{3+}) + {}^{4}F_{7/2}(Er^{3+}) \qquad \text{(S1-5)} $$

$$ 4f^{1}(Ce^{3+}) + hv(254\,nm) \xrightarrow{GSA} 5d^{1}(Ce^{3+}) \qquad \text{(S1-6)} $$

$$ 5d^{1}(Ce^{3+}) \rightarrow {}^{6}P_{7/2}(Gd^{3+}) + {}^{7}F_{0}(Eu^{3+}) \longrightarrow {}^{8}S_{7/2}(Gd^{3+}) + {}^{5}D_{4}(Eu^{3+}) \qquad \text{(S1-7)} $$

$$ {}^{4}F_{7/2}(Er^{3+}) \rightarrow {}^{2}H_{11/2}(Er^{3+}) \rightarrow {}^{4}I_{15/2}(Er^{3+}) + hv(520\,nm) \qquad \text{(S1-8)} $$

$$ {}^{4}F_{7/2}(Er^{3+}) \rightarrow {}^{4}S_{3/2}(Er^{3+}) \rightarrow {}^{4}I_{15/2}(Er^{3+}) + hv(540\,nm) \qquad \text{(S1-9)} $$

$$ {}^{5}D_{4}(Eu^{3+}) \rightarrow {}^{5}D_{0}(Eu^{3+}) \rightarrow {}^{7}F_{6}(Eu^{3+}) + hv(590\,nm) \qquad \text{(S1-10)} $$

$$ {}^{5}D_{4}(Eu^{3+}) \rightarrow {}^{5}D_{0}(Eu^{3+}) \rightarrow {}^{7}F_{2}(Eu^{3+}) + hv(615\,nm) \qquad \text{(S1-11)} $$

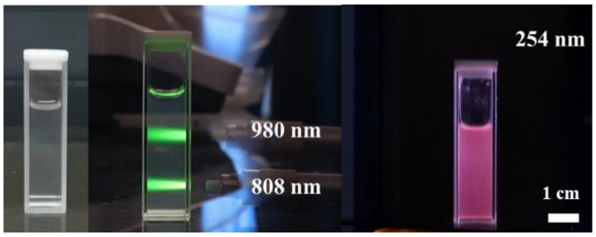

Figure S15 Er@Nd@Eu dispersion was stimulated by 980, 808, 254 nm to emit light.

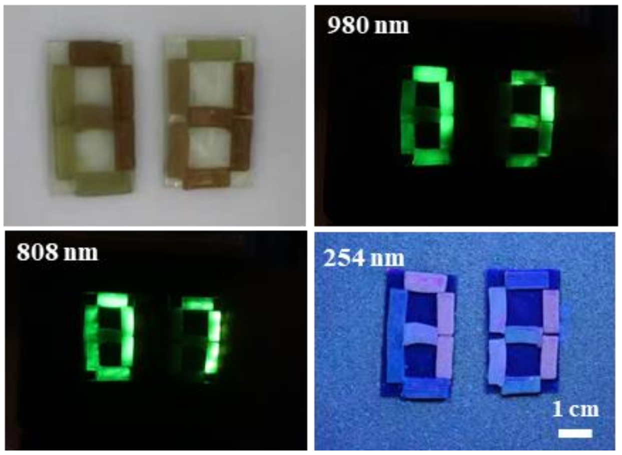

Figure S16 3D printed encoding device display different digital information under different excitation lights.

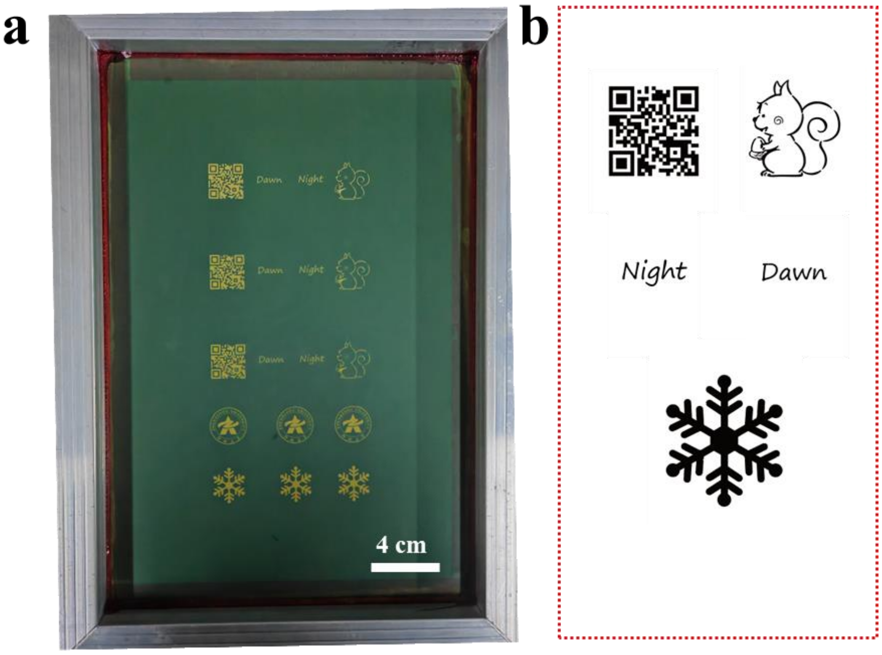

Figure S17 (a) Screen printing plate. (b) Screen print pattern.
